# Artificial Intelligence in Breast Cancer Diagnosis and Treatment: Advances in Imaging, Pathology, and Personalized Care

**DOI:** 10.3390/life14111451

**Published:** 2024-11-08

**Authors:** Petar Uchikov, Usman Khalid, Granit Harris Dedaj-Salad, Dibya Ghale, Harney Rajadurai, Maria Kraeva, Krasimir Kraev, Bozhidar Hristov, Mladen Doykov, Vanya Mitova, Maria Bozhkova, Stoyan Markov, Pavel Stanchev

**Affiliations:** 1 Department of Special Surgery, Faculty of Medicine, Medical University of Plovdiv, 4002 Plovdiv, Bulgaria; puchikov@yahoo.com; 2 Faculty of Medicine, Medical University of Plovdiv, 4000 Plovdiv, Bulgaria; usmankhalid957@gmail.com (U.K.); ghds2000@gmail.com (G.H.D.-S.); ghaledibya@gmail.com (D.G.); harney06@gmail.com (H.R.); 3 Department of Otorhinolaryngology, Medical Faculty, Medical University of Plovdiv, 4002 Plovdiv, Bulgaria; kraevamaria93@gmail.com (M.K.); stoyan_bgus2002@yahoo.com (S.M.); 4 Department of Propedeutics of Internal Diseases, Medical Faculty, Medical University of Plovdiv, 4002 Plovdiv, Bulgaria; 5 Second Department of Internal Diseases, Section “Gastroenterology”, Medical Faculty, Medical University of Plovdiv, 4002 Plovdiv, Bulgaria; hristov.bozhidar@abv.bg; 6 Department of Urology and General Medicine, Medical Faculty, Medical University of Plovdiv, 4001 Plovdiv, Bulgaria; mladen.doykov@mu-plovdiv.bg; 7 University Specialized Hospital for Active Oncology Treatment “Prof. Ivan Chernozemsky”, 1756 Sofia, Bulgaria; vania_mitova@mail.bg; 8 Medical College, Medical University of Plovdiv, 4000 Plovdiv, Bulgaria; mariya.bozhkova@mu-plovdiv.bg; 9 Clinic of Endocrinology and Metabolic Diseases, St George University Hospital, Medical University of Plovdiv, 4002 Plovdiv, Bulgaria; pavel.stanchev@mu-plovdiv.bg

**Keywords:** breast cancer, artificial intelligence, pathology, imaging

## Abstract

Breast cancer is the most prevalent cancer worldwide, affecting both low- and middle-income countries, with a growing number of cases. In 2024, about 310,720 women in the U.S. are projected to receive an invasive breast cancer diagnosis, alongside 56,500 cases of ductal carcinoma in situ (DCIS). Breast cancer occurs in every country of the world in women at any age after puberty but with increasing rates in later life. About 65% of women with the *BRCA1* and 45% with the *BRCA2* gene variants develop breast cancer by age 70. While these genes account for 5% of breast cancers, their prevalence is higher in certain populations. Advances in early detection, personalised medicine, and AI-driven diagnostics are improving outcomes by enabling a more precise analysis, reducing recurrence, and minimising treatment side effects. Our paper aims to explore the vast applications of artificial intelligence within the diagnosis and treatment of breast cancer and how these advancements can contribute to elevating patient care as well as discussing the potential drawbacks of such integrations into modern medicine. We structured our paper as a non-systematic review and utilised Google Scholar and PubMed databases to review literature regarding the incorporation of AI in the diagnosis and treatment of non-palpable breast masses. AI is revolutionising breast cancer management by enhancing imaging, pathology, and personalised treatment. In imaging, AI can improve the detection of cancer in mammography, MRIs, and ultrasounds, rivalling expert radiologists in accuracy. In pathology, AI enhances biomarker detection, improving *HER2* and *Ki67* assessments. Personalised medicine benefits from AI’s predictive power, aiding risk stratification and treatment response. AI also shows promise in triple-negative breast cancer management, offering better prognosis and subtype classification. However, challenges include data variability, ethical concerns, and real-world validation. Despite limitations, AI integration offers significant potential in improving breast cancer diagnosis, prognosis, and treatment outcomes.

## 1. Introduction

Breast cancer is the most prevalent cancer worldwide, affecting both low- and middle-income countries, with a growing number of cases. In 2024, about 310,720 women in the U.S. are projected to receive an invasive breast cancer diagnosis, alongside 56,500 cases of ductal carcinoma in situ (DCIS). A large majority of these invasive cases (84%) and deaths (91%) affect women aged 50 and above, with nearly half (52%) of all fatalities occurring in those aged 70 and older. The breast’s composition, which includes adipose tissue, glandular structures, blood vessels, and lymphatic networks, is influenced by hormonal changes. These hormonal fluctuations, tied to the menstrual cycle, may contribute to patterns of breast cancer development and progression, highlighting the role of both anatomy and hormonal regulation in epidemiological trends [1,2].

*BRCA1* and *BRCA2* are genes strongly linked to breast cancer, primarily due to their roles in DNA damage repair. Variants of these genes significantly increase the risk of breast and ovarian cancers. A study combining data from 22 investigations found that by age 70, 65% of women with *BRCA1* and 45% with *BRCA2* variants develop breast cancer. While these genes account for about 5% of all breast cancers, their prevalence is higher in specific populations, such as Ashkenazi Jewish women. Over 2000 variants of *BRCA1* and *BRCA2* exist, with some posing a higher cancer risk depending on their location in the gene [3,4].

The World Health Organization (WHO) emphasises that improving breast cancer outcomes and survival rates hinges on early detection, which is the cornerstone of breast cancer management strategies [5]. Breast cancer diagnosis and treatment are evolving from a uniform approach to personalised medicine. Advanced diagnostics, such as molecular imaging and genomic profiling, allow for more precise tumour analysis. These tools, along with innovative surgical methods and radiation therapies, contribute to a multidisciplinary strategy aimed at reducing recurrence and minimising the side effects of treatment [6].

Due to the vast and varied nature of imaging data, artificial intelligence (AI) has become a valuable tool for automatically analysing radiographic and pathological patterns, aiding in the early detection, precise diagnosis, and better prognosis of breast cancer [7]. AI also facilitates the integration of various data sources, such as medical images and biological records, into diagnostic systems to advance precision medicine. Intelligent systems are anticipated to enhance diagnostic accuracy and consistency, while easing radiologists’ workload and minimising patient complications from overdiagnosis [8].

Our paper aims to explore the vast applications of artificial intelligence within the diagnosis and treatment of breast cancer and how these advancements can contribute to elevating patient care as well as discussing the potential drawbacks of such integrations into modern medicine.

## 2. Methodology

**Figure 1:**
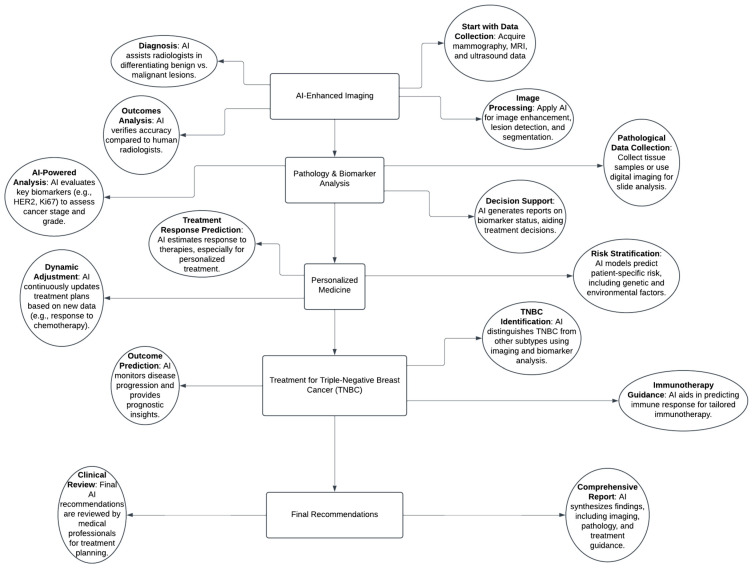
This demonstrates a flowchart highlighting the directions for using AI in diagnosing and treating breast cancer.

We structured our paper as a non-systematic review and utilised the Google Scholar and PubMed databases to review the literature regarding the incorporation of AI into the diagnosis and treatment of non-palpable breast masses (Figure 1). We used a combination of keywords and phrases to identify articles which consisted of neural network, artificial intelligence, non-palpable breast cancer, diagnostics and treatment, MRI, mammography, ultrasound, HER2, BRCA1, BRCA2, triple-negative breast cancer. Articles related to the key words were the basis of our search composition. We included articles which were published up to August 2024. Articles which were not published in English were excluded from the criteria.

Inclusion Criteria:

1. Articles related to artificial intelligence in breast cancer diagnostics.
2. Original articles of full-text length, covering the diagnoses, treatment plans, and results of breast cancer.
3. Review papers and abstract.

We opted towards a non-systematic review for the following reasons:

1. AI in healthcare is an expansive area. A non-systematic review allows for a broader exploration of recent developments, studies, and diverse methodologies that might not meet the strict criteria for a systematic review.
2. With a combination of clinical studies, case reports, and theoretical discussions on AI applications. A non-systematic review can include these varied sources to provide a comprehensive view of the current source material.
3. Non-systematic reviews can integrate expert opinions, clinical experiences, and theoretical frameworks, which are valuable for understanding the implications of AI technologies in practice, especially in complex clinical contexts like breast masses.

## 3. Results

### 3.1. AI-Enhanced Imaging: Mammography, MRI, and Ultrasound

The role of imaging in breast cancer diagnostics is crucial. Still, with the increase in AI integration and utilisation, there remains room to profit from the use of AI to enhance the diagnosis of breast cancer (BC). The integration of AI into imaging diagnostics for BC may allow for a quantitative representation of medical images which can assist with the diagnosis and prognosis of BC. Furthermore, AI improves the quality of images from data augmentation, leading to a faster detection/segmentation of breast lesions and diagnosing malignant lesions [8].

In the last 30 years, BC screening with mammography has been implemented across a broad range of countries. With time, the use of computer-aided detection (CADe) and diagnosis (CADx) was introduced in response to digitization and considerable interest in the use of computer-assisted image interpretation. However, improvements in clinical outcomes for image analysis were not met and a considerable number of false positives were seen. On the other hand, within recent years, an increased utilisation of AI and deep learning (DL) has revolutionised the capabilities of automated detection of breast cancer from mammography interpretation. In recent years, studies have shown that AI’s performance is on par with that of an experienced breast radiologist in retrospective datasets [9]. A study conducted by Dembrower et al. (2023) [10] which assessed the routine mammography results of 55,581 women aged between 40 and 74 at a hospital in Stockholm, Sweden, showed that 0.5% of the women were diagnosed with breast cancer based on an initial positive read from screen detection and double reading of this was demonstrated by one radiologist and AI. It showed that the AI was not inferior to the double reading from two radiologists without AI. The confidence interval (CI) shown here was between 1.00 and 1.09. Furthermore, the comparison of triple reading utilising two radiologists and AI with a double reading by two radiologists was also non-inferior with a relative proportion of 1.08. This demonstrates that the potential integration of AI can be carried out under controlled measures with a comparison to real-world performance follow-up [10].

In relation to MRI, the incorporation of AI has the potential to heighten the diagnostic capabilities of doctors as well as to accurately determine the tumour volume, the phenotypic characteristics of the tumour, and the prediction of risk associated with the tumour. Breast MRIs making use of AI have provided an opportunity for further integration of multiple data streams for use in multidisciplinary teams (MDTs) when it comes to the determination of patient-centred therapy [11].

Research performed by Jiang et al. (2021) aims to demonstrate whether there is any benefit from using AI to differentiate cancerous cells from non-cancerous cells in breast dynamic contrast material-enhanced (DCE) MRI with the utilisation of software already readily available. The imaging conducted was interpreted by a group of 19 radiologists specialising in breast imaging from a variety of academic and private sectors. They interpreted the images in two attempts, on the first attempt, utilising the readily available existing software and on the second attempt with assistance from AI. Upon a thorough evaluation of the reader diagnostic performance, it was found that the area under the curve (AUC) of all the interpreters improved from 0.71 to 0.76 when AI systems were used. The sensitivity of the AI-interpreted results was improved when the cut-off point was Breast Imaging Reporting and Data System (BI-RADS) Category 3 with a confidence interval (CI) for change of 0.8% to 7.4%, but this was not the case for Category 4 where the confidence interval was −0.9% to 11%. On the other hand, the average specificity between categories 4a and 3, when either was used as a cut-off point, showed no considerable difference with the following results obtained: 52% and 52% [95% CI: −7.3%, 6.0%], and from 29% to 28% [95% CI: −6.4%, 4.3%]. The study concluded that the use of systems integrated with AI elevated the abilities of the radiologists to distinguish between benign and malignant breast lesions on an MRI [12]. An important indicator related to the treatment and survival prediction of breast cancer patients is associated with sentinel lymph node metastasis (SLNM). As per a study from Wang et al. (2023), the purpose of using this indicator is to assess whether deep learning (DL) models on various US modalities such as grayscale US, colour Doppler flow imaging (CDFI) US, and elastography imaging can be used to classify the SLNM in BC. The study assessed 317 BC patients over 2 years and 11 months. The data obtained were randomly allocated to either training cohorts or internal validation cohorts with a ratio of 7:3. In addition, an external validation cohort, utilising 42 patient datasets, was conducted at another affiliated hospital. The three DL models for each US modality mentioned above were compared and evaluated to determine their degree of diagnostic accuracy based on the area under the curve (AUC). It was found that, for the internal and external cohort evaluation of the Grayscale US DL, the AUC was 0.855 and 0.788, respectively. Overall, it was determined that the DL of the elastography US was far superior relative to the DL grayscale US and DL CFDI US. The AUC of the internal cohort for the DL elastography was 0.879 and the external cohort AUC of 0.876. The model for elastography also demonstrated a remarkable accuracy, sensitivity, and specificity in both cohorts. Therefore, DL elastography has been proven to demonstrate a superior diagnostic performance for management in patients with SLNM in BC [13]. This further emphasises the pivotal role that artificial intelligence and deep learning can have on the diagnosis and management of breast cancer, with the ability to dive deeper into the disease progression concerning metastasis and not just be limited to primary tumour detection.

AI-driven support systems enhance intraoperative decision-making and productivity through the enhancement of surgical margin visualisation. Levy et al. compiled scans from 151 patients through computationally efficient convolutional neural networks. The CNN model efficiently detects suspicious regions within surgical margins, achieving on-device inference times of around 10 milliseconds for a 420 × 2400 image. In independent tests on 155 pathology-confirmed margins, including 31 positive margins from 29 patients, the model delivered an AUROC of 0.976, with a sensitivity of 0.93 and a specificity of 0.98. At the margin level, the deep learning model accurately identified 96.8% of pathology-positive margins. These findings underscore the clinical potential of AI-enhanced margin visualisation using WF-OCT in breast cancer surgeries and its ability to reduce reoperation rates by detecting residual tumours [14]. Similar studies utilised ensemble methods to predict positive and close margins (tumour-to-margin distance ≤2.0 mm) in ultrasound images, leveraging eight pre-trained deep neural networks. The most effective ensemble approach for segmentation achieved a median Dice score of 0.88 on our dataset. By utilising these segmentation outcomes, we attained a sensitivity of 96% and a specificity of 76% for predicting close margins, as validated against histology results. These encouraging findings further reinforce the potential of AI-powered ultrasound imaging as a tool for intraoperative margin assessment during breast-conserving surgery [15].

### 3.2. AI in Pathology: Diagnostic Accuracy and Biomarker Evaluation

Pathology and biomarker detection in breast cancer (BC) is of paramount importance for diagnosis and management plans for BC. Typical biomarkers that are monitored/tested include *HER2*, progesterone receptor status, oestrogen receptor (OR) status, and *Ki67*. The utilisation of AI in the field of routine pathology has the potential to enhance diagnostic accuracy and help to reduce the occurrence of preventable errors (Table 1). In relation to research conducted by Wu et al. (2024), it has been shown that the use of AI can allow for increased accuracy and reproducibility concerning *HER2* receptor detection. Their study intended to demonstrate to what extent current AI algorithms can be used for the immunohistochemical diagnosis of HER2-positive breast lesions. They focused their work on seven studies which assessed 6867 cases. The studies utilised different AI algorithms, for instance; two studies used the HER2-CONNECT algorithm, two studies used the CNN algorithm, one study used the multi-class logistic regression algorithm, and a further two used the *HER2* 4B5 algorithm. The immunohistochemical assessment of HER2 breast cancer is graded in the following way: *HER2 0/1+*, *HER2 2+*, and *HER2 3+*. Their findings for each of these grades were as follows: For *HER2* 0/1+, the sensitivity of AI was 0.98 and the specificity was 0.92. For the *HER2* 2+, the sensitivity of AI was 0.78 and the specificity was 0.98. Finally, regarding *HER2* 3+, the AI sensitivity was found to be 0.99 and the specificity was 0.99. Despite the fact that that there are some differences in the results across the different immunohistochemical grading scales, the overall interpretation here is that AI shows considerable promise in the *HER2* status assessment of breast lesions [16].

In current medical practice, the detection of breast cancer markers primarily involves invasive methods. A meta-analysis study conducted by Fu et al. (2024) aimed to investigate to what extent AI can be incorporated into the detection/prediction of these markers in ultrasound-based radiomics. Their results yielded solid foundational data with regard to *HER2* prediction (specificity: 0.78, sensitivity: 0.76) and for *Ki67* prediction (specificity: 0.76, sensitivity: 0.80). On the other hand, with regard to the prediction of the ER and progesterone receptor (PR), there were insufficient data for quantitative analysis. Therefore, the accuracy of AI in *Ki67* and *HER2* prediction can be inferred [24]. AI has the potential to alleviate a variety of obstacles in the pathological diagnosis of BC with a heightened ability to provide accurate diagnoses by reducing the inter-observer variability in the interpretation, leading to faster-reviewing speeds and identifying poorly obtained slides. AI can commit to this by considering a variety of components including nutritional/genetic/environmental factors as well as calculating individual risk assessments aiding the course of diagnosis and the frequency of monitoring. The abilities of AI within this field of diagnosis are of crucial importance since it can aid in valuable decision-making for clinicians and further enhance treatment protocols for respective patients [25].

Over the years, enhancements and progressions made in the field of technology have allowed for an increased utilisation of digitalised whole-slide images with slide scanners. In response to this, considerable efforts are being made to form algorithms with the capabilities to automatically analyse digital pathological images and in turn, determine disease severity. Introducing and incorporating AI into this field will lead to a considerable reduction in diagnosis time and reduce inter- and intra-observer variability. Such functionalities cannot gain success without sufficient data input from a wide range of readily available sources as well as different types of staining and cutting procedures for biopsies. Cruz-Roa et al. (2017) suggested training classifiers with 400 exemplars from a variety of sources and further directly assessing and validating a sample of 200 cases directly from the Cancer Genome Atlas concerning invasive BC. They found a Dice coefficient of 75.86% with positivity of 71.62% and negativity of 96.77% in response to a thorough pixel evaluation of these images in comparison to the manually annotated regions. The high Dice coefficient of these results demonstrates that there is a strong association between the algorithm predictions and the manual annotations, outlining that the AI has the capability to replicate the work of the pathologist in identifying areas with tumour activity [17]. McCaffrey et al. (2024) conducted a study regarding the various benefits of using AI in digital pathology. The incorporation of digital methods into BC pathology involves both AI-based algorithms and a whole-slide image scanner with which the combined data are quantified. There is potential for game-changing protocols in BC diagnosis with the potential for AI to detect biomarkers from tumour microenvironments simply from digital image assessments. This provides essential practical implications with a focus on increasing the speed of diagnosis and the accuracy of diagnosis and increasing the speed of treatment initiation. Expert evaluation of the study highlighted the heightened contributions that AI can make regarding the analysis of the tumour microenvironment and bio-marker detection as well as its overall involvement in diagnosis and prognosis [26]. By using AI in the pathology of BC diagnosis, a variety of hurdles can be overcome, all of which have the potential to enhance patient outcomes. A faster pathological/biomarker assessment incorporating AI into digital image analysis also potentially leads to a potentially higher diagnostic accuracy. This in turn decreases the diagnosis-to-treatment period and provides valuable treatment guiding information. This in turn can have an overwhelming influence on the treatment protocol for patients and potentially increase the likelihood of favourable outcomes [26].

### 3.3. Personalised Medicine: Risk Stratification and Treatment Response Prediction

Personalised medicine has shown the potential to enhance the clinical outcomes of patients. The involvement of AI in personalised medicine has shown promise in determining the appropriate treatment protocol as well as predicting treatment outcomes. In recent years, considerable strides have been made in the development of ways to utilise the full or near-full potential of AI which have led to innovation and an acceleration in the development of ways to guide patient tailored management. A wide range of studies have shown that AI can be used in various ways within the healthcare system to aid the management of breast cancer. The approaches utilising radiomics and radio-genomics have the potential to identify the genomics characteristics of breast lesions to promote targeted treatment [23]. In recent years, AI has become increasingly accurate in predicting 5-year survival rates in patients with BC. Wang et al. has followed up on 604 breast cancer patients who were diagnosed and underwent standard treatment between 2000 and 2003 at a hospital in Taiwan. The artificial neural network utilised STATISTICA software v.12.0. For the AI to predict the 5-year survival of the patients, various factors were considered including age, type of surgery, type of radiotherapy, size of the tumour, and presence or absence of regional lymph node/distant metastasis. The results found an astounding 85% accuracy rate of the AI in predicting the 5-year survival rate in BC patients with an area under the curve of 0.79. This demonstrates that AI can be an essential tool to guide clinicians in determining the 5-year survival likelihood of their patients [18].

Personalised medical treatments are constantly developing and strengthening in the field of BC as well as general oncology. Therapies are constantly being adapted to individual needs and tumour genetics, as well as other characteristics of the tumour. These advancements, as mentioned, may prove to be integral for the treatment outcomes of patients, providing the possibility of a reduced cancer-associated mortality and an overall better prognosis. Sohrabei et al. assessed and analysed the results from 46 studies which involved patients on personalised BC treatment regimens developed with the involvement of AI. Seventeen of the studies that analysed the data focused specifically on multiple deep learning methods and yielded satisfactory results for predicting the survival of patients and classifying their breast lesions. Two of the studies that they assessed used neural networks and clustering, which proved to be promising in terms of predicting the survival of patients. Twenty-six of the studies that they analysed used machine learning methods, and these had the most promising results as, in this regard, the AI was most effective at classifying, diagnosing, and determining the prognosis of the disease. The models showed a 90–96% accuracy, sensitivity, specificity, and precision. This study employed the use of various modelling techniques including NB, SVM, RF, XGBoost, and Reinforcement Learning. All of the components and results assessed demonstrate that AI can be remarkably effective in helping to assist researchers and clinicians to dive deeper into the full picture of a breast lesion, incorporating genetics and other complex information that may otherwise be missed. Such advancements may prove to be pivotal for disease management, particularly for individualised treatment protocols [19]. A study conducted by Skarping et al. (2022) aimed to prove that an AI analysis of digital mammograms can be used to predict the response of the patient to neoadjuvant chemotherapy (NACT). The study involved a sample of 453 patients who received NACT between 2005 and 2019. The deep learning system used for the mammogram analysis was the baseline digital mammogram, and it made a prediction of the pathological complete response of the tumour after NACT. In total, 95 of the patients in the study achieved a complete pathological response. The average age of the patients was 52.5 years, and 255 were premenopausal. The results regarding the prediction models set out by the AI were shown to have an AUC of 0.71 (95% confidence interval 0.53–0.90; *p* = 0.035). The sensitivity demonstrated by the AI was 46% and the specificity was 90%. This allows us to infer that the clinical decisions and treatment regimens that are given to a patient can be aided by the utilisation of AI algorithms [27].

Another considerable challenge is the fact that breast cancer can have a broad range of molecular aetiologies as well as a wide range of clinical outcomes depending on the subtype in question. The utilisation of AI and large data algorithms can promote individualised and faster diagnosis and treatment options for BC. The stratification of BC is a crucial aspect of effective treatment as well as individualised treatment options. The increased incorporation of Support Vector Machine (SVM) has proven to be a key to the success of AI as it offers the opportunity to rearrange large datasets into smaller datasets called support vectors, which helps to speed up the time needed for data analysis. This can prove to be crucial for a wide scope of BCs, especially those with a more aggressive biological behaviour. The SVM can initially be used to subtype the BC as well as analyse specific characteristics of the breast lesion; following this, it can be used to create models of effective therapy which clinicians can take into consideration for better treatment outcomes tailored to the patient [22].

An additional innovation within personalised medicine lies in the use of natural language processors and deep learning methods to develop a Breast Cancer Risk Calculator (BRISK). This calculator aims to reduce over diagnosis and excessive biopsies. In their study, He et al. utilised 5000 patient records from Houston Methodist (2006–2015). By integrating imaging, pathology, and demographic data to provide refined biopsy recommendation index for BI-RADS 4 patients, BRISK achieved a 100% sensitivity and 74% specificity, with an overall accuracy of 81% and an AUC of 0.93, improving upon traditional BI-RADS scoring. This tool supports more informed biopsy decisions, with further prospective evaluations underway [28]. Similar studies using an intelligent BRISK software achieved a high predictive accuracy for BI-RADS 4 malignancies, with an accuracy of 89.5%, AUC of 0.93 (95% CI: 0.92–0.95), sensitivity of 100%, and specificity of 81%, based on a dataset of 4209 women (median age 56). Among 1228 patients in the “low” probability of malignancy (POM) group, only 0.16% had malignancies, while the “high” POM group had an 85.9% malignancy rate. As a continuous malignancy predictor, the iBRISK score reached an AUC of 0.97 (95% CI: 0.97–0.98), with potential cost savings of over USD 420 million by potentially avoiding biopsies for up to 50% of low-to-moderate POM patients [29]. 

### 3.4. Innovations in Triple-Negative Breast Cancer Management

Triple-negative breast cancer (TNBC) accounts for 10–15% of all invasive breast cancer subtypes [30]. AI can provide important molecular and clinical data to treat a malignancy that lacks effective therapeutic targets, is highly invasive, and has a high recurrence rate. It improves the accuracy of TNBC diagnosis and the classification of subtypes. Therefore, AI can also be applied to tailor treatment and provide a prognosis.

Tumour-infiltrating lymphocytes (TILs) have emerged as predictive and prognostic biomarkers for patients with TNBC. Biomarkers of tumour receptors, such as TILs, are used to diagnose TNBC as their evaluation presents a greater reproducibility. Machine learning and computational pathology advances show great promise as they enable TILs to be assessed computationally. By utilising automated image analysis, these new advancements provide a more standardised and reliable evaluation compared to traditional visual TIL assessment (VTA). The automation of image analysis addresses some of the challenges associated with manual assessments such as subjectivity and variability, enhancing the accuracy and efficiency of TIL evaluations [31]. In addition to the immunogenic microenvironment, tumour–stroma interactions are clinically significant and typically analysed through histopathology. The stroma surrounding tumour tissue carries a prognostic value, as patients with stroma-high tumours show a worse prognosis compared with women with a low tumour–stroma ratio. Modern AI and deep learning-based image analysis allow for the digitisation of tissue slides into whole-slide images, making it possible to observe tumour–stroma interactions and study the spatial tumour microenvironment. Deep convolutional neural networks have become the foremost branch of AI for image analysis, showing the ability to classify and segment tissue types and different kinds of nuclei effectively and accurately. This creates new opportunities to understand the spatial tumour microenvironment [20].

There is a need in TNBC to develop effective predictive and prognostic biomarkers to help stratify and personalise patient management. Typically, radiological scans and histopathological tissue samples have been analysed by physicians to diagnose and guide the treatment of patients. However, there may be disease-specific information that is not easily perceived by the human eye and may be missed. This is where the application of AI can be helpful, as it can be used for diagnostic imaging assessment and digitised histopathological sampling of tissues. This carries the potential for risk stratifying patients to identify those more likely to experience disease recurrence or die from the disease and predict pathologic complete response [21]. Within the tumour microenvironment, the invasion of surrounding tissue results in clusters or single tumour cells called tumour budding, reflecting tumour progression. The host also attempts to establish antitumour immunity by increasing tertiary lymphoid structures. Integrating an analysis of both TB and TLS can help predict patient prognosis as the elements of the dynamic relationship between the tumour and immune system are studied. Generally, the assessment of TB and TLS relies on histopathological analysis, but this approach is limited by inter-observer variation because of manual and qualitative evaluations, this is both time-consuming and labour-intensive. AI-adopted methods such as automated imaging offer a more standardised solution and can also improve workflow efficiency, reduce workload, and enhance overall performance. The results from the study demonstrated a positive correlation between the TLS/TB index and the overall survival and relapse-free survival of patients with TNBC [32].

Conventional cytotoxic chemotherapy is the usual treatment for patients in both early and advanced stages of the disease, showing significant benefits in neoadjuvant, adjuvant, and metastatic settings [33]. However, recent advancements in cancer immunotherapy with immune checkpoint inhibitors show promise. A study conducted by Garrone et al. (2024) on the use of immunotherapy in melanoma patients can allow us to build a foundation for developing effective immunotherapies for TNBC using AI. It suggests that immunotherapy can induce a durable response in patients with metastatic cancers compared to conventional therapies. Some immunotherapies can allow the immune system to recognise and attack cancer cells by targeting immune checkpoints. This results in longer-lasting responses or, in some patients, complete remission [34]. A study by Boulenger et al. in 2023 presents the development of a deep learning-based system capable of automatically distinguishing TNBC from other molecular subtypes using only ultrasound images. In clinical practice, surgical resection determines the molecular subtype with certainty as core biopsy samples may not fully represent the entire lesion, particularly in heterogeneous tumours. The study highlights the use of non-invasive methods as histopathological sampling can be invasive. Ultrasound imaging, known for its convenience and high sensitivity to breast nodules in dense breast tissue, is the basis for this model. This model enables accurate preoperative prognosis predictions, facilitating more precise and comprehensive treatment decisions. The model also automatically distinguishes TNBC from other molecular subtypes such as luminal A, luminal B, and *HER2*-positive cancer. Based on a total of 145 female breast cancer patients without a breast cancer history who underwent ultrasound examination by a single radiologist, the model achieved a sensitivity of 85.7% and specificity of 86.3% [35].

A study conducted by Yang et al. introduces an advanced deep learning (DL) model that integrates multi-omics data to improve the accuracy of subtype classification and prognosis prediction in triple-negative breast cancer (TNBC), addressing challenges associated with its aggressive nature. Using comprehensive breast cancer data, the model demonstrates the significant advantages of multi-omics integration. By applying Bayesian optimisation, the DL model structure is refined to optimise predictive power. The results show that the multi-omics DL model outperformed the single-omics models, achieving accuracies of 98% in cross-validation, 97% in the validation set, and 91% in an external test set. An MRI-based radiomics model also demonstrated strong initial performance, but its cross-dataset accuracy decreased, underscoring the robustness of the multi-omics DL model in providing consistent and clinically relevant predictions across data sources [36]. 

A separate study explored an AI-powered approach to drug discovery that integrates OMICS data—specifically transcriptomics and gene profiles—to target triple-negative breast cancer (TNBC) through pyroptosis, a form of programmed cell death. Using deep learning on extensive molecular datasets, the researchers identified and optimised drug combinations designed to activate pyroptosis-related pathways in TNBC cells. Their analysis showed significant differential expression in the transcriptome profiles of 360 TNBC tissues compared to 88 normal breast tissues, with many genes exhibiting notable upregulation. A relational map of 45 genes revealed extensive interactions among them, indicating that targeting pyroptosis may be a promising treatment strategy. Notably, the AI model demonstrated a high predictive accuracy in selecting effective drug pairs, outperforming traditional methods. This work highlights the potential of integrating OMICS data with AI to enhance precision therapy for challenging cancers like TNBC [37]. 

### 3.5. Issues with Implementation

Breast cancer is a leading cause of cancer mortality among women; therefore, AI is being increasingly used to support more accurate diagnoses and effective treatment. AI uses multiple technologies and applications, like deep learning with convolutional neural networks and transfer learning (TL). TL allows learned features to be applied to new tasks with limited data, reducing the need for extensive training. However, there can be obstacles like dependence on operator-related factors and equipment quality, affecting static ultrasound image quality. Variability can also be based on the region of interest selected for a lesion by the radiologist. Furthermore, existing AI systems fail to utilise additional diagnostic tools beyond 2D imaging, such as cine loops, elastography, and colour Doppler. AI typically evaluates each lesion in isolation without considering the existence of other lesions within the breast, which can impact diagnostic accuracy. Other limitations include high costs, inconsistent performance, and concerns over reliability, bias, and ethical implications [38]. Studies have also lacked sufficient data for a strong quantitative analysis particularly in oestrogen and progesterone receptor markers, affecting result reliability, research by Fu et al. found that only three studies retrieved patients from multiple data centres, which does not reflect the characteristics of the entire population and data collected retrospectively can cause high potential of bias causing an issue with AI integration [24].

AI algorithms are susceptible to biases embedded within healthcare data, which can influence their outcomes. Alongside well-recognised biases in research, such as those in sampling and blinding, it is crucial to recognise both implicit and explicit biases inherent in the healthcare system. Large datasets used to train AI models may reflect these biases, affecting the predictions the algorithms make. Factors like clinical trial eligibility criteria and subtle biases in treatment decisions can shape AI-driven clinical recommendations. Furthermore, AI may contribute to healthcare disparities through biased data collection practices, algorithmic design, insufficient diversity in training datasets, and a lack of transparency within research teams. Addressing these issues is essential to ensure fair outcomes. The adverse effects of model bias on demographic characteristics, including sex and ethnicity, are increasingly recognised. Studies have also highlighted lower implementation rates for certain health interventions among rural populations, racial and ethnic minority groups, the uninsured or underinsured, and individuals with lower income or educational attainment, underscoring the need for equity-focused AI in healthcare [39,40].

Different systems can be used in AI for breast cancer from using it in biomarkers to density measurement assessment tools such as Volpara or CNN models. The challenges faced with the innovation of AI are clinical validation and, again, the ability to ensure it works effectively in real life settings rather than just in controlled studies, there is also an issue in terms of the AI models’ ability to perform consistently across many different datasets, being able to acquire diverse data and ensuring models help in disease management, as AI model creation is based on data from the majority population, the minority population may be misrepresented. AI systems can be referred to as ‘black boxes’ as it can be unclear how they make the decisions and there is cost of the development and ongoing maintenance of the systems [41]. While it may seem that using the same AI tool across all doctors creates a sense of standardisation, a significant drawback is the limited understanding of how AI arrives at its conclusions within the black box problem. Although AI models train and appear to refine their outputs over time, users often lack insight into the specific steps or data the AI employed to reach its final recommendations. The individuals relying on the AI are unaware of which factors were prioritised, undervalued, or potentially excluded. Even if this information was accessible, interpreting it would be incredibly difficult since the most advanced AI models today process billions of parameters simultaneously. This lack of transparency presents a unique challenge in assessing whether human biases have influenced AI algorithms [42]. AI analysis of mammography has shown great potential. Saliency maps study the roles of breast lesions in breast cancer detection. Saliency analysis demonstrated its efficacy in a study performed by Pertuz et al. on mammograms collected from 191 individuals, including those with breast cancer and healthy controls. However, AI systems can have performance issues. Research indicates that AI often underperforms when validated independently with a drop in the AUC of 0.256 to 0.361 using external data, indicating a reduced effectiveness, likely due to differences in test populations and experimental design such as variations in age, population demographics, and previous cancer history. AI systems sometimes show a low amount of overlap between AI areas of interest and actual lesions with higher-performance systems, such as End2End, which had a low overlap but performed better overall in detecting breast lesions. While interpretability-focused systems, such as GMIC and GLAM, aimed at being clearer in targeting specific regions in mammograms, showed lower accuracy and missed detecting lesions, suggesting that enhancing interpretability might reduce the effectiveness of the detection of breast cancer using AI. For example, saliency analysis sometimes lacks correlation in how accurate the AI’s prediction is, as the decision-making process is not fully explained. Although 179 works used XAI, there was only one that had measures to evaluate it. This means future research needs larger datasets to include more detailed clinical and imaging information [43].

When considering the legislation in place for data and privacy protection, the General Data Protection Regulation (GDPR), introduced by the European Union, set a new standard for privacy laws globally, influencing legislation beyond Europe, including in countries like the United States and Canada. GDPR mandates that any personal data or activities related to foreign individuals or companies under its jurisdiction must be processed by a data controller or processor within the EU to ensure the robust protection of individual privacy. In the United States, the Genetic Information Nondiscrimination Act (GINA) prevents employers from making discriminatory decisions based on a person’s genetic information, safeguarding individuals’ genetic privacy in employment and health insurance contexts. Meanwhile, artificial intelligence (AI) is transforming healthcare by analysing health data, interpreting medical imaging, and contributing to research advancements, all aimed at enhancing diagnostic accuracy and treatment outcomes. Social media platforms also play an essential role in public health, often sharing timely health news and information, especially during global health crises. However, this increasing reliance on AI and data-sharing platforms raises significant privacy concerns, particularly regarding patient data security. Current regulations in healthcare fall short of comprehensively safeguarding personal health information. Patient data collected by AI-powered devices and robots is at risk of security breaches, exposing sensitive information to malicious actors. Moreover, some social media platforms collect extensive data on users, including mental health information, often without explicit consent, which can be exploited for advertising and marketing purposes. Additionally, certain genetic testing and bioinformatics companies, which may lack proper oversight, have been known to sell customer data to pharmaceutical and biotech firms [44,45].

In research conducted by McCaffery et al., they reviewed studies of different models as well as some challenges; these can be split into key criteria such as challenges with algorithms or software. Algorithm challenges can be due to data limitations, validation required for real-world use, and issues with transparency or interpretability again referred to as “black box”. These can be issues for both deep learning and machine learning models. Issues with the software can include the elevated cost of the necessary technology, as AI needs powerful computers which have a GPU (specialised graphic cards). The storing of large amounts of digital images, required for AI systems can also be costly, this is alongside the difficulty in receiving financial support as well the limit in insurance reimbursement of AI-related expenses. For example, in a study by Phan et al. Several models were tested on 138 patients to predict recurrence, which showed ranging accuracies of 84–91%. These are good indications but can show that the variability can be due to inconsistent data or limited sample sizes [26]. There can also be chances of false-positive findings such as in convolutional CADSs, computer-aided detection/diagnosis systems, where around 1000 prompts must be analysed to detect additional cancer which can result in increased rates of biopsy or no benefit in diagnosis accuracy [8].

Recent public–private partnerships aimed at implementing machine learning in healthcare have raised significant concerns regarding patient privacy. A notable example is the 2016 collaboration between DeepMind, a subsidiary of Alphabet Inc. (Google), Mountain View, CA, USA, and the Royal Free London NHS Foundation Trust to use machine learning for managing acute kidney injury. Critics highlighted that patients lacked control over how their data were used and that the implications for privacy were not sufficiently addressed. A senior advisor from England’s Department of Health even remarked that the patient data were obtained through an “inappropriate legal basis” [46]. The situation escalated when Google took direct control of DeepMind’s application, effectively shifting the oversight of patient data from the UK to the US. This ability to transfer vast amounts of sensitive patient information across jurisdictions exemplifies a troubling aspect of big data, particularly in the context of commercial healthcare AI, where public institutions may become overly dependent on tech companies, leading to a power imbalance [47]. Although some breaches of patient privacy may have occurred despite existing laws and regulations, the DeepMind case underscores the urgent need for robust safeguards to protect privacy and uphold patient autonomy in public–private partnerships. AI presents unique challenges, as algorithms typically require extensive access to patient data, which may be utilised in various ways over time. The location and ownership of the servers housing these data are crucial considerations, prompting a call for regulations that ensure patient information remains within its jurisdiction, barring exceptional circumstances. Strong privacy protections can be achieved when institutions are inherently designed to promote collaboration for data security. While the integration of healthcare AI can be managed to safeguard privacy, it often leads to conflicting interests, as corporations may prioritise data monetization over patient privacy unless sufficiently incentivized to protect it. Given these challenges, there are increasing demands for the enhanced oversight of big data in health research and technology [48].

Within the realm of pathology, specifically whole-slide imaging, an array of problems are presented with the integration of AI into diagnostic procedures. Technical challenges are the most common, such as scan failures requiring re-scans, which extend turnaround times (TATs). The longer digital evaluation times reported by pathologists further risk delaying TATs, though this may be offset by DP’s workflow benefits, including faster image access and easier archival retrieval. Newer scanners, uniform histological preparations, and z-stacking for cytology digitization help reduce these issues. Validation studies also highlight diagnostic challenges in specific tasks, like grading dysplasia and counting mitoses, with accuracy discordance reported in areas like gastrointestinal dysplasia and meningiomas. AI-driven tools show promise in addressing these challenges by enhancing dysplasia grading, mitotic counts, and microorganism detection. Additionally, storage and IT demands, as well as some pathologists’ resistance to DP, have presented obstacles, though regulatory approvals and remote work needs during COVID-19 have bolstered confidence in WSI. Ongoing improvements in imaging technology and AI support continue to advance DP’s role in reliable histological diagnoses for adults and paediatric cases alike [49].

With the integration of AI in breast cancer, there can be difficulty adapting to the newer imaging techniques such as digital breast tomosynthesis (DBT) where challenges can occur due to the difficult nature of 3D imaging. Although AI presents benefits in the form of a reduced workload and improved accuracy, a study conducted by Schaffter et al. showed that no single AI model outperformed radiologists. However, with the use of an ensemble of AI models and radiologists, there was an improvement in mammography accuracy. This highlights the importance of radiologists and AI collaboration [23]. The results Schaffter et al. reported used 31 different AI digital mammography (DM) algorithms and were evaluated using two datasets, one with over 40,000 patients from the US and the other with 166,000 patients from Sweden [9].

In high-stakes decisions impacting an individual’s life, such as choosing to withdraw care or proceed with a high-risk procedure, a responsible party has traditionally been accountable for the final call. Today, when patient autonomy is limited due to lack of capacity, this responsibility lies with the treating physician, often in consultation with psychiatric experts, service chiefs, and ethics committees. However, if AI algorithms were to take on this role, addressing adverse outcomes would become more complex, underscoring the need for guidelines on when physicians might “overrule” an AI’s recommendation. While giving physicians the authority to override AI decisions might seem prudent, it introduces its own challenges; if an AI is proven to perform specific tasks more accurately and efficiently than humans, physician intervention could inadvertently harm patients. Ignoring a robust AI model’s recommendation could be likened to disregarding the assessment of a radiologist or the direction of an attending physician, given the documented errors in human judgement and the limits of traditional clinical decision-making [50].

Given the early adoption of such technology, it is imperative that an overreliance on AI does not become normalised within medical care. Within Boulenger et al.’s study, multiple limitations were detected. First, the small sample size and the fact that data were obtained from a single centre highlight the need for validation of the model’s predictive capabilities using external datasets in a multi-centre context, which is crucial for clinical application. Second, the study involved collecting ultrasound images that captured at least the largest diameter sections and orthogonal views. However, it remains unclear whether the model’s predictive performance would be significantly impacted by including additional ultrasound images from a single index lesion. Third, while this research focuses on the binary classification of triple-negative cases, subsequent studies should explore the four-way molecular subtyping of breast cancer. Finally, benchmarking various deep learning models, including non-convolutional neural networks such as Vision Transformers, could aid in identifying the most effective architecture for the task and improving overall performance [35]. The nomogram-based prediction model presented similar limitations. First, the sample size utilised for developing this model was relatively small, highlighting the need for further validation in a larger cohort of patients with triple-negative breast cancer (TNBC). Second, the analysis was limited to tumour-infiltrating lymphocytes (TLSs) and tumour burden (TB) scores, restricting the range of variables considered within the tumour microenvironment (TME). Third, validation was conducted exclusively using data from the Affiliated Cancer Hospital of Tianjin Medical University, without incorporating data from other cancer centres, which would enhance the reliability of the findings. Lastly, the investigation focused solely on the correlation between the TLS/TB index and clinical outcomes for patients with TNBC, without examining other breast cancer subtypes [32].

AI and machine learning hold the potential to streamline and enhance clinical trials by improving participant matching, recruiting, and data analysis. They could also facilitate the creation of artificial control groups by comparing historical data to trial requirements and assist in predicting adverse events and patient subpopulations more accurately. Additionally, AI could generate “synthetic patients” to simulate diagnostic or treatment outcomes. However, implementing AI in clinical trials introduces uncertainties that must be carefully addressed in study design and reporting. The evaluation criteria traditionally applied in medicine, where progress often comes in the form of new treatments, are not yet fully defined for AI interventions. The medical community expects the same rigour in validating AI-based interventions as it does for medications. Key considerations include (1) addressing clinically relevant issues that impact provider decisions and improve outcomes, (2) ensuring scalability and applicability across diverse demographics and disease profiles, and (3) generating universally beneficial outcomes, beyond the specific training data. Furthermore, AI-based recommendations should consider public health impacts, including resource allocation, to meet ethical expectations in healthcare [51].

## 4. Conclusions

In conclusion, the integration of artificial intelligence into breast cancer diagnosis and treatment represents a significant leap forward in oncology. AI’s ability to process and analyse vast amounts of data allows for more precise and timely detection of breast cancer, enhancing diagnostic accuracy and leading to earlier intervention. By identifying patterns and anomalies that might elude human detection, AI systems improve the sensitivity and specificity of breast cancer diagnoses, contributing to better patient outcomes. Beyond diagnosis, AI’s influence extends to treatment planning and management. Machine learning algorithms can aid in personalising treatment strategies by analysing patient data and predicting responses to various therapies. This capability facilitates more tailored and effective treatment plans, potentially reducing side effects and improving overall efficacy. However, the integration of AI into breast cancer care is not without its challenges. Ensuring that these systems are dependable, generalizable across diverse patient populations, and free from biases is essential for equitable healthcare delivery. Additionally, ongoing validation and regulatory oversight are critical to maintain high standards in both diagnostic accuracy and treatment efficacy. In summary, the convergence of AI with breast cancer diagnosis and treatment heralds a new era of precision medicine. By continuing to advance research, addressing challenges, and fostering collaboration between technologists and healthcare providers, we can harness AI’s full potential to enhance diagnostic and therapeutic outcomes, advancing the quality of care for breast cancer patients.

## Figures and Tables

**Table 1:** The below demonstrates some of the key findings with specific AI algorithms arranged according to publication.

Publication	Aspect	AI Methodology	Key Findings
Dembrower et al. [10] Jiang et al. [12]	Imaging in Diagnostics	AI-enhanced mammography, MRI, ultrasound, CADe, CADx	AI enables early detection and accurate diagnosis, rivalling expert radiologists in image interpretation. Studies show AI reduces false positives and improves sensitivity in mammography and MRI, with accuracy comparable to experienced radiologists.
Levy et al. [14] Veluponnar et al. [15]	Intraoperative Margin Assessment	CNN, ultrasound segmentation, WF-OCT	AI algorithms, such as convolutional neural networks (CNNs), enhance surgical accuracy by identifying cancerous margins quickly, reducing the need for reoperations. WF-OCT models yield an AUROC of 0.976 with a high sensitivity (0.93) and specificity (0.98).
Wu et al. [16] Cruz-Roa et al. [17]	Pathology and Biomarker Analysis	CNN, HER2-CONNECT, multi-class logistic regression	AI aids in biomarker detection, improving the accuracy of HER2, ER, PR, and Ki67 readings and enhancing the reproducibility of these assessments across diverse samples, addressing inter-observer variability.
Wang et al. [18] Sohrabei et al. [19]	Personalised Medicine	Neural networks, clustering, SVM, RF, XGBoost, deep learning	AI facilitates risk stratification and treatment prediction, particularly in estimating 5-year survival rates and predicting treatment responses. Personalised medicine using AI improves outcomes by tailoring treatments based on genetic and phenotypic characteristics.
Albusayli et al. [20] Corredor et al. [21]	Triple-Negative Breast Cancer	Machine learning for TIL and stroma analysis	AI-based image analysis of tumour-infiltrating lymphocytes (TILs) and tumour–stroma interactions provides insights into prognosis and treatment, particularly useful in TNBC where traditional biomarkers are less effective. Studies show significant prognostic value in determining the tumour–stroma ratio and TIL assessment.
Ozer et al. [22] Cè et al. [23]	Risk Stratification and Subtyping	SVM, radio-genomics, computational pathology	Machine learning algorithms such as SVM facilitate breast cancer subtyping and stratification, allowing tailored treatment regimens based on specific subtypes and individual characteristics. AI-based subtyping shows a high accuracy for aggressive and diverse cancers.
Fu et al. [24] Singh et al. [25]	Non-invasive Prediction of Biomarkers	Ultrasound-based AI, radiomics	AI-driven ultrasound radiomics predict key biomarkers like HER2 and Ki67 with a high specificity and sensitivity. This non-invasive approach offers reliable options for biomarker analysis, reducing dependency on biopsy procedures.
